# Beyond Weight Loss: A Comprehensive Review of Pregnancy Management following Bariatric Procedures

**DOI:** 10.3390/medicina60040635

**Published:** 2024-04-15

**Authors:** Iulia Huluță, Livia-Mihaela Apostol, Radu Botezatu, Anca Maria Panaitescu, Corina Gică, Romina-Marina Sima, Nicolae Gică, Florina Mihaela Nedelea

**Affiliations:** 1 Clinical Hospital of Obstetrics and Gynaecology Filantropia, 011132 Bucharest, Romania; iuliahuluta16@gmail.com (I.H.); livia-mihaela.cosma@rez.umfcd.ro (L.-M.A.); radu.botezatu@umfcd.ro (R.B.); anca.panaitescu@umfcd.ro (A.M.P.); mat.corina@gmail.com (C.G.); romina.sima@umfcd.ro (R.-M.S.);; 2 Obstetrics and Gynecology Department, Carol Davila University of Medicine and Pharmacy, 050474 Bucharest, Romania

**Keywords:** gastric bypass, micronutrient deficiencies, glucose metabolism, folic acid

## Abstract

The increasing prevalence of bariatric surgery among women of childbearing age raises critical questions about the correct management of pregnancy following these procedures. This literature review delves into the multifaceted considerations surrounding pregnancy after bariatric surgery, with a particular focus on the importance of preconception counselling, appropriate nutrition assessment, and the necessity of correct folic acid supplementation. Key areas of investigation include nutrient absorption challenges, weight gain during pregnancy, and potential micronutrient deficiencies. Examining the relationship between bariatric surgery and birth defects, particularly heart and musculoskeletal issues, uncovers a twofold increase in risk for women who underwent surgery before pregnancy, with the risk emphasized before folic acid fortification. In contrast, a nationwide study suggests that infants born to mothers with bariatric surgery exhibit a reduced risk of major birth defects, potentially associated with improved glucose metabolism. In addition, this review outlines strategies for managing gestational diabetes and other pregnancy-related complications in individuals with a history of bariatric surgery. By synthesizing existing literature, this paper aims to provide healthcare providers with a comprehensive framework for the correct management of pregnancy in this unique patient population, promoting the health and well-being of both mother and child.

## 1. Introduction

The World Health Organization (WHO) reports that in recent years, obesity has reached alarming levels worldwide, leading to a variety of health complications and a decrease in quality of life for those who are affected [1]. Bariatric procedures, such as gastric bypass and sleeve gastrectomy, have emerged as effective interventions for significant and sustained weight loss [2]. Extensive research has been conducted on the impact of these procedures on weight loss, comorbidities, and long-term health outcomes. However, despite the increasing number of women of childbearing age opting for bariatric surgery, there remains a dearth of information regarding the management of pregnancy following these procedures [3]. This article aims to provide a comprehensive review of the management of pregnancy after bariatric procedures, exploring the impact on fertility, maternal health, fetal development, and the long-term implications for both current and future pregnancies.

## 2. Bariatric Procedures: A Brief Overview

Bariatric procedures are surgical interventions specifically designed to address weight loss in individuals with obesity. The three most frequently performed procedures are gastric bypass, gastric banding, and sleeve gastrectomy, which are briefly summarized in Table 1 [4]. Each of these procedures brings about anatomical modifications to the digestive system, resulting in reduced calorie intake, malabsorption, or a combination of both mechanisms [5].

The primary goal of bariatric procedures is to achieve significant and sustained weight loss. The amount of weight loss varies depending on the procedure and individual factors. Gastric bypass and sleeve gastrectomy generally result in more substantial weight loss compared to gastric banding [6]. Bariatric procedures have profound effects on metabolism. They often lead to improved insulin sensitivity and glucose control, reducing the risk of obesity-related conditions such as type 2 diabetes [7,8]. These metabolic changes contribute to overall improved health and well-being. They can impact nutrient absorption and nutrient deficiencies. For instance, gastric bypass and sleeve gastrectomy can result in malabsorption of certain nutrients, such as iron, vitamin B12, calcium, and folate [9]. Regular monitoring of nutrient levels and appropriate supplementation are crucial to avoid deficiencies [10]. Bariatric procedures alter the digestive system, potentially leading to changes in gastrointestinal function [11]. These changes may include changes in bowel habits, increased risk of gallstones, and alterations in gut hormones that control hunger and satiety. Patients should be aware of these potential effects and receive appropriate postoperative care [10].

## 3. Bariatric Procedures and Pregnancy 

### Endocrine and Reproductive Parameters

Obesity is a known risk factor for infertility and can negatively impact reproductive outcomes. It is associated with menstrual irregularities, hormonal imbalances, anovulation (lack of ovulation), and decreased responsiveness to fertility treatments [12,13]. Weight loss has been shown to have a significant positive effect on fertility rates, and bariatric surgery offers a promising intervention for individuals struggling with obesity and infertility [13]. 

As bariatric surgery has emerged as an effective treatment for weight loss, it has been found to have a positive impact on fertility in women [14,15]. Several mechanisms contribute to the improvement in fertility following bariatric procedures, and they are summarised in Table 2. 

Studies have reported increased rates of spontaneous conception following bariatric procedures, indicating improved fertility outcomes in women who have undergone surgery. For example, a meta-analysis of 13 studies found that bariatric surgery was associated with higher rates of spontaneous conception, with a significant increase in live birth rates compared to non-surgical weight loss interventions [15,22].

Gray et al. evaluated the effects of bariatric surgery on fertility in both males and females by analyzing changes in various parameters such as sex hormone levels, sperm parameters, menstrual irregularities, and sexual function. As a result of the analysis, testosterone (TT), free testosterone (FT), follicle-stimulating hormone (FSH), luteinizing hormone (LH), and sex hormone-binding globulin (SHBG) levels significantly improved in males one year after surgery, with a concurrent decrease in estradiol levels. Erectile function also showed a substantial increase at the 12-month follow-up. However, sperm parameters did not show significant changes except for sperm morphology. The study attributed these improvements to the substantial loss of adiposity post-surgery, which reduced aromatase activity, improved insulin sensitivity, and regulated sex hormone levels, ultimately leading to enhanced spermatogenesis. Studies, however, have indicated that sperm parameters may have worsened as a result of nutritional deficiencies that commonly occur following bariatric surgery.

In contrast, in obese females, bariatric surgery led to a significant decrease in testosterone levels but increased levels of LH, FSH, and SHBG, along with lower estradiol levels. Following surgery, the FSFI scores indicated improved sexual function [23]. Menstrual irregularities decreased significantly at the 6-month follow-up, but did not change significantly at the 12-month follow-up. The reduction in testosterone levels post-surgery was attributed to increased synthesis of SHBG and improved insulin sensitivity, leading to decreased ovarian androgen production [23]. They demonstrate the importance and even cruciality of bariatric surgery in obese men and women who struggle with reproductive health, especially when finding it difficult to lose and maintain weight [23].

## 4. Preconception Counselling

In order to achieve a successful pregnancy after bariatric surgery, preconception counselling is essential. A contraceptive strategy should be emphasized during the weight loss phase to prevent unwanted pregnancies. Timing to conceive after bariatric surgery is an important consideration for individuals who are planning to become pregnant. Although bariatric surgery can improve ovulation rates, it is recommended to wait before attempting pregnancy to allow for optimal weight stabilization and minimize potential risks to the developing fetus. Despite the fact that studies indicate there is no significant difference in birth weight between babies born to women who conceived within a year after bariatric surgery and those who waited more than a year, there are differing recommendations from different professional bodies regarding the ideal duration to wait before getting pregnant [24].

The American Association of Clinical Endocrinologists, the Obesity Society, and the American Society for Metabolic & Bariatric Surgery recommend avoiding conception for 12–18 months after bariatric surgery [10]. The American College of Obstetricians and Gynecologists advises women to wait between 12–24 months to give birth, whereas the Royal College of Obstetricians and Gynecologists recommends a flexible approach based upon the woman’s age and discourages older women from attempting to delay pregnancy [25].

The type of bariatric surgery also plays a role in determining the appropriate timing to conceive. Studies have shown that small-for-gestational-age babies are more commonly associated with women who have undergone procedures like Roux-en-Y gastric bypass or adjustable gastric banding. In these cases, it is recommended to wait for twelve to eighteen months post-surgery before attempting pregnancy. A retrospective case-control study of 150 pregnancies occurring within the first 18 months after laparoscopic sleeve gastrectomy did not find an increase in adverse outcomes [26].

Note that, especially in the patient who has been lost to follow-up after the initial one to two years post-surgery, there may be a higher risk of underlying micronutrient deficiencies [27]. Therefore, it is crucial to check nutrient levels and provide appropriate supplementation before attempting pregnancy in individuals with a longer interval between surgery and pregnancy [27].

In the latest guidelines of the European Society of Endoscopic Surgery, it is recommended to postpone pregnancy during weight loss after bariatric surgery. This is also supported by lower admission fees to child care centers at younger ages, thus reducing the risk of adverse outcomes for the fetus [28].

During preconception counselling, women should be educated about the need for adequate nutrient intake, including proper vitamin and mineral supplementation. Folic acid, in particular, is critical for preventing neural tube defects in the developing fetus. Additionally, counselling should address the potential risks associated with pregnancy following bariatric surgery, such as the increased risk of small-for-gestational-age infants and preterm birth. 

## 5. Maternal Health

### 5.1. Gestational Weight Gain 

Pregnancy following bariatric surgery requires special attention to gestational weight gain to ensure optimal maternal and fetal health. While some weight gain during pregnancy is necessary for fetal growth and development, excessive weight gain can lead to increased risks of gestational diabetes, preeclampsia, and caesarean delivery [29]. Women who underwent adjustable gastric banding or sleeve gastrectomy may experience a slower rate of weight gain during pregnancy, while those who underwent Roux-en-Y gastric bypass may experience a more rapid rate of gain [4,6,30]. The appropriate amount of gestational weight gain varies depending on pre-pregnancy BMI and the type of bariatric surgery performed (see Table 3). In addition to the significance of monitoring BMI, there is growing recognition of the influence of gestational weight gain on pregnancy outcomes [31]. The consensus regarding optimal gestational weight gain is primarily based on the guidelines established by the Institute of Medicine (IOM). According to these guidelines, pregnant women with a normal BMI (18.5–24.9 kg/m^2^), overweight BMI (25–29.9 kg/m^2^), and obese BMI (≥30 kg/m^2^) are advised to gain 11.5–16 kg, 7–11.5 kg, and 5–9 kg, respectively [32].

### 5.2. Increasing Need for Nutrients during Pregnancy 

Bariatric surgery can impact the absorption of key nutrients, including iron, vitamin B12, calcium, and folate, which are essential for maternal and fetal health. During pregnancy, the demand for these nutrients increases, and it may be challenging for women who have undergone bariatric surgery to meet their increased nutrient needs [34].

To avoid nutrient deficiencies, regular monitoring of nutrient levels and appropriate supplementation is crucial. Over-supplementation of certain nutrients should also be avoided as it can lead to toxicity. Therefore, tailored supplementation is recommended based on the individual’s specific needs and health status.

All guidelines universally recommend the use of prenatal multivitamin and mineral supplementation both before conception and throughout pregnancy. Specifically, for women who have undergone bariatric surgery and are planning a pregnancy or are already pregnant, it is strongly advised to conduct a biochemical assessment to determine their specific micronutrient requirements. The recommended supplement should include, at a minimum, the following micronutrients: copper (2 mg), zinc (15 mg), selenium (50 μg), folic acid (0.4–1 mg), iron (45–60 mg or >18 mg for those with an adjustable gastric band), thiamine (>12 mg), vitamin E (15 mg), and beta-carotene (vitamin A, 5000 IU) [35]. Regarding folic acid supplementation, current guidelines recommend a daily dose of 4 or 5 mg for women who remain obese or diabetic after bariatric surgery during the preconception period and throughout the first trimester [36]. For vitamin B12 supplementation, it is suggested to administer a dose of 1 mg every 3 months via intramuscular depot injection during the preconception period, and monthly throughout pregnancy [36,37]. Alternatively, oral supplementation (1 mg/day) can be used to enhance patient compliance [37]. In line with all guidelines for women who have undergone bariatric surgery and are pregnant, it is recommended to supplement with a minimum daily dose of 45 mg of elemental iron (>18 mg for those with an adjustable gastric band). This dosage should be adjusted as necessary to maintain normal ferritin levels. If oral supplementation is deemed insufficient, clinical recommendations suggest considering iron infusion [10]. To maintain a serum concentration of 50 nmol/L or above of vitamin D and ensure that serum parathyroid hormone remains within normal limits, vitamin D supplementation is recommended. Furthermore, calcium supplementation may be added if necessary to maintain parathyroid hormone within normal limits [38].

For comprehensive prenatal monitoring, it is recommended to assess various indices at least once per trimester, utilizing pregnancy-specific reference ranges. These indices include but are not limited to serum folate, serum vitamin B12, iron studies (including serum ferritin and transferrin saturation), full blood count, serum vitamin D (along with calcium, phosphate, magnesium, and parathyroid hormone), serum vitamin A, prothrombin time, International Normalized Ratio (INR), serum vitamin K1 concentration, serum protein and albumin levels, as well as renal and liver function tests [36]. 

### 5.3. Insulin Resistance and Gestational Diabetes

Insulin resistance is a common metabolic disorder associated with obesity and type 2 diabetes. Bariatric surgery has been shown to improve insulin resistance and glucose metabolism and prevent the development of type 2 diabetes [39]. The risk of gestational diabetes, which is associated with insulin resistance, is also reduced after bariatric surgery.

Murine research points to a dynamic interplay between the gut, brain, and liver, potentially influencing glucose regulation. The intricate roles of gastrointestinal hormones in the remission of surgically induced diabetes remain incompletely understood [40]. In human studies, alterations in GLP-1 and PYY release patterns have been linked to changes in food intake, gluconeogenesis, and weight loss in the initial months post-surgery. Enhanced GLP-1 responses correlated with improved β-cell function in the early postoperative period. Notably, recent research underscores the significant contribution of the PYY hormone to long-term improvements in glucose-mediated insulin and glucagon secretion among bariatric patients. Furthermore, the beneficial metabolic outcomes of bariatric surgery may stem from alterations in circulating adipokines and hepatocytes, such as adiponectin, retinol-binding protein 4 (RBP4), fibroblast growth factor-21 (FGF21), and C-reactive protein (CRP). These molecules possess anti-inflammatory, insulin-sensitizing, and lipid-lowering properties. The mechanisms underlying changes in their secretion are likely multifaceted, involving substantial weight reduction and weight-loss-independent factors [41].

Several studies have investigated the effects of bariatric surgery on GDM risk of gestational diabetes. These studies have demonstrated that surgery can reduce the rate of gestational diabetes development in pregnant women with a history of obesity, particularly those who have undergone Roux-en-Y gastric bypass (RYGB) [42].

Numerous studies have consistently indicated that bariatric surgery significantly reduces the risk of gestational diabetes mellitus (GDM) compared to preoperative levels or obese controls. While certain studies have highlighted a reduction in GDM risk post-surgery to levels comparable with the general population [43,44,45], others have shown a continued elevated risk compared to normal weight controls [46,47,48]. Notably, the decrease in GDM rates persists in subsequent pregnancies following bariatric surgery [49]. A recent systematic review matching participants to pre-bariatric surgery weight demonstrated a significant reduction in GDM risk post-surgery, with an odds ratio of 0.21 (95% CI 0.11–0.37) and a number needed to benefit of 5 [50]. However, when women were matched for pre-pregnancy BMI, the association between bariatric surgery and decreased GDM risk was no longer observed [50], suggesting that weight loss primarily drives the reduced risk rather than specific postoperative mechanisms. Evidence also indicates that the interval from surgery to conception does not notably affect GDM rates, with similar reductions observed during and after the first year post-surgery [51,52]. In summary, bariatric surgery emerges as an effective strategy to mitigate GDM risk in obese women. Nevertheless, given that a substantial portion of post-bariatric surgery women remain obese, their overall risk of GDM tends to exceed that of normal-weight pregnant women [53]. Consequently, screening for GDM should be standard practice for all pregnant women who have undergone bariatric surgery [53].

In conclusion, bariatric surgery can have significant metabolic and endocrine effects and improve insulin sensitivity, glucose control, and lipid profiles, thereby reducing the risk of gestational diabetes. Close monitoring of blood glucose levels, thyroid function, and other metabolic and endocrine parameters is necessary to ensure healthy pregnancy and optimal outcomes.

## 6. Management of Pregnancy following Bariatric Surgery 

Pregnancy following bariatric surgery requires careful management to mitigate potential risks and optimise outcomes for both the mother and the fetus. To ensure the best possible outcomes, preconception counselling, regular prenatal care, and multidisciplinary collaboration are crucial aspects of managing pregnancies in individuals who have undergone bariatric procedures.

### 6.1. Regular Prenatal Care

Regular prenatal care is essential for monitoring the progress of pregnancy and ensuring the well-being of both the mother and the fetus. Close monitoring of weight gain is particularly important, as inadequate weight gain or excessive weight gain can have adverse effects on maternal and fetal health. Healthcare providers should individualize weight gain recommendations based on pre-pregnancy body mass index and the specific bariatric surgery procedure undergone, as discussed in the Gestational Weight Gain Section [30].

Apart from weight, nutrient levels and hormonal status should also be closely monitored throughout pregnancy. This includes regular assessments of vitamin and mineral levels, particularly folic acid and vitamin B12. The availability of nutrients is essential for supporting fetal development and avoiding complications. Hormonal assessments, such as monitoring thyroid function, should be conducted to ensure hormonal balance [35].

Given the unique healthcare needs of women who have undergone bariatric surgery during pregnancy, a multidisciplinary approach involving collaboration between bariatric and obstetric teams is recommended. This collaboration allows for comprehensive care that addresses specific concerns related to both the bariatric procedure and the pregnancy. The bariatric team can provide expertise in managing nutrition and identifying potential nutrient deficiencies, while the obstetric team can address specific concerns related to pregnancy and fetal development. This collaboration ensures that women receive comprehensive care, with appropriate monitoring and interventions tailored to their individual needs [51].

### 6.2. Fetal Development and Neonatal Outcomes

Bariatric surgery has been associated with beneficial effects on fetal development and neonatal outcomes, such as reductions in gestational diabetes, large-for-gestational-age infants, and neonatal complications. The improvements in maternal metabolic profiles and the reduction in maternal obesity-related inflammation are believed to contribute to these positive outcomes. However, it is important to note that some studies have suggested a potential association between bariatric surgery and an increased risk of small-for-gestational-age infants and preterm birth [54]. This indicates the need for close monitoring of fetal growth and development throughout pregnancy to ensure appropriate fetal growth and well-being.

There have been conflicting findings regarding the potential association between bariatric surgery and the risk of birth defects. While several case reports have suggested a possible link [43,44,45,46], retrospective studies have mostly found no significant association [55,56,57,58,59,60]. Additionally, it remains unclear whether the increased risk observed in some studies is solely attributed to bariatric surgery or if obesity, which is also a risk factor for birth defects, plays a role. A study involving 1845 deliveries among women who had bariatric surgery before pregnancy found that bariatric surgery was associated with a 1.20-fold higher risk of birth defects in subsequent pregnancies, compared to those who had no surgery or were obese. In contrast, obesity without bariatric surgery was only weakly associated with birth defects. The association between bariatric surgery and birth defects was more pronounced for heart and musculoskeletal defects. Before folic acid food fortification, bariatric surgery was linked with birth defects, whereas after fortification, this association diminished. Specifically, compared to individuals with no surgery or obesity, those who underwent bariatric surgery before fortification faced a 2.03-fold higher risk of birth defects (95% CI: 1.41, 2.92). However, the risk was reduced substantially to 1.05-fold (95% CI: 0.86, 1.28) for those who had surgery after fortification. Interestingly, obesity alone, without surgery, was associated with birth defects after fortification (RR: 1.13; 95% CI: 1.09, 1.17). Notably, having bariatric surgery after pregnancy showed no association with birth defects in either period. [61] In addition to close monitoring, proper vitamin intake, including folic acid and vitamin B12, plays a crucial role in supporting fetal development and reducing the risk of certain complications. Adequate folic acid intake is essential for preventing neural tube defects in the developing fetus. Vitamin B12 is important for neurological development and is particularly important for vegetarians or individuals who have undergone malabsorptive procedures like Roux-en-Y gastric bypass, as they may be at risk of vitamin B12 deficiency.

### 6.3. Delivery and the Postpartum Period

The mode of delivery in individuals who have undergone bariatric surgery is typically not contraindicated for vaginal delivery, unless specific obstetric factors are present, such as placenta praevia, macrosomia, or abnormal fetal positioning. Previous studies have reported conflicting findings regarding the rate of cesarean section in individuals who have had bariatric surgery. A systematic review by Vrebosch et al. found a lower rate of cesarean section in this group [62], while a meta-analysis by Yi et al. found no significant difference in cesarean delivery rates [52].

Breastfeeding is an important aspect of newborn care and offers numerous health benefits to both the mother and the baby. In a two-year prospective study published in the Journal of Nutrition, researchers analyzed breast milk samples from 86 participants across two Belgian hospitals to assess macronutrients and vitamin A concentration. Samples were collected from the third day post-delivery until the sixth week of lactation. Among the participants, 24 were of normal weight, 39 were overweight, 12 were obese, and 11 had undergone bariatric surgery, aged between 29 and 3. The study included an evaluation of maternal diets and physical activities during the six-week postpartum period through questionnaires.

Results indicated that bariatric surgery did not alter breast milk composition. Interestingly, women who had undergone bariatric surgery exhibited higher fat, milk energy, and carbohydrate levels during the initial two weeks compared to normal-weight, overweight, and obese counterparts. Protein and vitamin A concentrations were initially lower but normalized in subsequent weeks.

Furthermore, all macronutrients were consistently higher in women with bariatric surgery compared to other weight categories. Notably, maternal diets showed no significant influence on breast milk composition changes, suggesting a potential impact on micronutrient levels instead [63]. Therefore, it is recommended that women who have had bariatric surgery continue to take nutritional supplements during the breastfeeding period and aim to breastfeed for at least six months after delivery [64].

## 7. Conclusions

As the prevalence of obesity continues to rise, bariatric procedures have become increasingly prevalent as effective weight-loss interventions. However, the focus on pregnancy outcomes following bariatric surgery remains limited. This comprehensive review aimed to bridge the gap by exploring the impact of bariatric procedures on fertility, maternal health, fetal development, and the long-term implications for both current and future pregnancies. By understanding the effects of bariatric surgery on pregnancy, healthcare professionals can provide appropriate guidance and optimal care to women considering or undergoing bariatric procedures. Further research and long-term follow-up studies are warranted to provide evidence-based guidelines for managing pregnancies following bariatric procedures.

## Figures and Tables

**Table 1:** Types of Bariatric Procedures [4,5,6].

Procedure	Description
Gastric Bypass	Also known as Roux-en-Y gastric bypass, it is one of the most common bariatric procedures performed. It involves the creation of a small stomach pouch and rerouting a segment of the small intestine to connect to the pouch. This procedure restricts food intake and alters the digestion and absorption of nutrients.
Gastric Banding	Involves placing an adjustable band around the upper part of the stomach, creating a small pouch. The band can be tightened or loosened to control the passage of food. This procedure restricts the size of the stomach and limits food intake.
Sleeve Gastrectomy	Also known as gastric sleeve surgery, it involves removing a significant portion of the stomach to create a smaller, sleeve-shaped stomach. This procedure reduces the capacity of the stomach and decreases the production of hunger-stimulating hormones.
Biliopancreatic Diversion (BPD)	Creates a smaller stomach (similar to gastric bypass surgery), but in addition, there is less absorption of ingested food inside the intestine (malabsorption).

**Table 2:** Mechanisms involved in the improvement of fertility following bariatric procedures.

Mechanism	Details
Hormonal changes	Bariatric surgery can lead to changes in hormonal profiles, including decreased insulin resistance, increased insulin sensitivity, and improvements in oestrogen and progesterone levels [16,17,18,19]. These hormonal changes can promote menstrual regularity, restore ovulation, and increase the chances of spontaneous conception [20,21].
Weight reduction	Significant weight loss following bariatric surgery can help restore normal hormonal functioning and menstrual regularity. Obesity-related hormonal imbalances can disrupt the menstrual cycle and inhibit ovulation. By reducing excess weight, bariatric surgery improves hormonal balance and restores ovarian function, increasing the likelihood of conception [20,21].
Reduced inflammation	Obesity is associated with a chronic state of inflammation, which can negatively affect reproductive function. Bariatric surgery has been shown to reduce systemic inflammation and improve markers of oxidative stress, which may create a more optimal environment for conception and pregnancy [19,21].
Improved insulin resistance	Obesity is often characterized by insulin resistance, which can lead to metabolic dysfunction and hormonal imbalances. Bariatric surgery improves insulin sensitivity and glucose control, which can positively impact fertility outcomes. Improved insulin sensitivity reduces the levels of circulating insulin and may help normalize hormone levels, leading to more regular menstrual cycles and improved reproductive function [20,21].

**Table 3:** Gestational weight gain depending on pre-pregnancy BMI and the type of bariatric surgery performed [33].

Type of Bariatric Surgery	Pre-Pregnancy BMI	Gestational Weight Gain
Adjustable Gastric Banding (AGB)	Less than 30 kg/m^2^	12–18 kg
	30 kg/m^2^–40 kg/m^2^	5–9 kg
	More than 40 kg/m^2^	2–5 kg
Roux-en-Y Gastric Bypass (RYGB)	Less than 30 kg/m^2^	11–20 kg
	30 kg/m^2^–40 kg/m^2^	5–9 kg
	More than 40 kg/m^2^	2–5 kg
Sleeve Gastrectomy (SG)	Less than 30 kg/m^2^	9–14 kg
	30 kg/m^2^–40 kg/m^2^	5–9 kg
	More than 40 kg/m^2^	2–5 kg
